# Inhibition of Adipose Tissue Lipolysis Treats Obesity-Related HFpEF

**DOI:** 10.1161/CIRCRESAHA.125.326251

**Published:** 2025-05-05

**Authors:** Alina Stockner, Maria Degli Innocenti, Daksh Verma, Sophie T. Schmid, Alexander Fuerlinger, Anila Varghese, Andreas Zirlik, Renate Schreiber, Rolf Breinbauer, Mahmoud Abdellatif, Simon Sedej

**Affiliations:** Department of Cardiology, Medical University of Graz, Austria (A.S., D.V., S.T. Schmid, A.F., A.V., A.Z., M.A., S. Sedej).; Institute of Organic Chemistry, Graz University of Technology, Austria (M.D.I., R.B.).; BioTechMed Graz, Austria (R.S., R.B., M.A., S. Sedej).; Institute of Molecular Biosciences, University of Graz, Austria (R.S.).; Faculty of Medicine, University of Maribor, Slovenia (S. Sedej).

**Keywords:** adipose tissue, caloric restriction, inflammation, lipolysis, obesity

Obesity is a major driver for the development and progression of heart failure with preserved ejection fraction (HFpEF), a multiorgan syndrome with growing prevalence and limited evidence-based therapies.^1^ Caloric restriction (CR) and new incretin-based therapies are powerful anti-obesity interventions that have shown promise in treating HFpEF.^2^ However, direct targeting of adipose tissue (AT) can offer a more selective and potentially safer alternative treatment option as it can mitigate the systemic side effects of CR and available HFpEF pharmacotherapies, which do not act solely nor directly on AT. Indeed, AT plays a central role in HFpEF by perpetuating chronic low-grade systemic inflammation,^3^ but currently there is no evidence that modulating AT metabolism is sufficient to treat obesity-related HFpEF. Here, we tested whether specifically inhibiting AT lipolysis through blocking its rate-limiting enzyme, adipose tissue triglyceride lipase (ATGL), improves obesity-related inflammation, cardiac remodeling, and dysfunction in HFpEF.

To elucidate whether genetic inhibition of ATGL in adipocytes is sufficient to protect from HFpEF, we subjected 7-week-old adipocyte-specific *Atgl* knockout mice (hereafter referred to as AAKO mice) and their control littermates to a high-fat diet and the nitric oxide synthase inhibitor, L-NAME (N[ω]-nitro-l-arginine methyl ester), for 15 weeks (Figure [A]). We used only male mice as females exhibit protection against HFpEF induced by high-fat diet+L-NAME.^4^ Animal experiments were conducted according to European ethical guidelines (Directive 2010/63/EU) and approved by the Austrian authorities (BMBWF-2022-0.061.336; 2022-0.824.559; 2023-0.862.526; 2023-0.336.345). AAKO-HFpEF mice exhibited resistance to gain body weight and had significantly reduced adiposity, measured by gonadal white AT (gWAT) mass, compared with control littermates-HFpEF mice (Figure [A]), despite similar food intake (AAKO-HFpEF, 2.59 g±0.16 g/mouse per day versus control littermates-HFpEF, 2.88 g±0.14 g/mouse per day; mean±SEM). All groups showed preserved ejection fraction (Figure [A]). Unlike control littermates-HFpEF mice, however, AAKO-HFpEF and AAKO-Chow mice displayed normal echocardiography-derived E/e′ ratio, a measure of diastolic function, and no left ventricular hypertrophy, as measured by tibia length-normalized heart weight (Figure [A]). These findings indicate that inhibiting AT lipolysis effectively prevents the development of obesity-related HFpEF.

**Figure.**
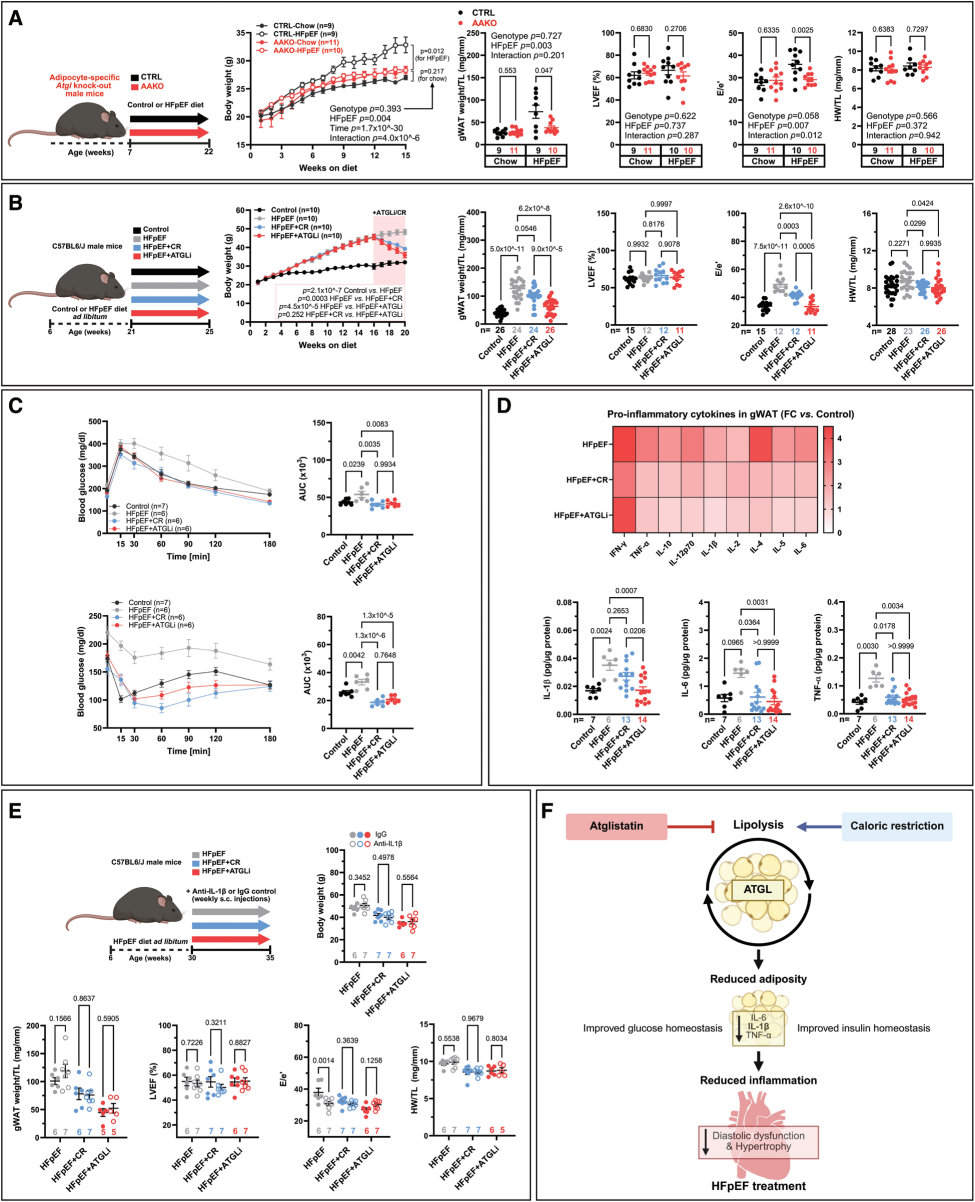
**Inhibition of adipose tissue lipolysis mitigates cardiometabolic heart failure with preserved ejection fraction (HFpEF). A** (**from left to right**), Schematic representation of the 2-hit HFpEF protocol in adipocyte-specific *Atgl* knockout (AAKO; *Pnpla2^flox/flox^-AdipoQ-Cre^+/-^*). AAKO mice and their control littermates (CTRL; *Pnpla2*^*flox/flox*^*-AdipoQ-Cre*^*-/*-^, all on C57BL6/J background) were fed a chow diet (Sniff, #EF D12450J) or an HFpEF-inducing diet, consisting of high-fat diet (HFD; 45% kcal from fat; Sniff, #EF D12451) and L-NAME (N[ω]-nitro-l-arginine methyl ester) (0.5 g/L in the drinking water) for 15 consecutive weeks. Body weight gain and gonadal white adipose tissue (gWAT) weight normalized to tibia length (TL). Echocardiography-based assessment of left ventricular ejection fraction (LVEF), and ratio of peak early Doppler transmitral flow velocity to myocardial tissue Doppler velocity (E/e’). Heart weight normalized to tibia length (HW/TL). **B** (**from left to right**), Schematic representation of the 2-hit HFpEF protocol in C57BL/6JRj mice fed standard or HFpEF diet ad libitum for 15 weeks. Obese mice with HFpEF were then randomly assigned to 3 weight-matched groups, of which a group continued receiving the HFpEF diet ad libitum for another 4 weeks (red area). The second group received atglistatin (ATGLi; 2 mmol/kg diet), and the third calorically restricted (CR) group had their daily food intake matched to the low amount consumed by the ATGLi group. Food was provided in partial allotments spread throughout the day to avoid extended daily periods of food intake restriction. The healthy control group continuously received a standard diet only. Body weight gain, gWAT weight/TL, LVEF, E/e’, and HW/TL. **C** (**top**), Blood glucose concentrations (**left**) and area under the curve (AUC; **right**) of intraperitoneal glucose tolerance test (GTT). **Bottom**, blood glucose concentrations (**left**) and AUC (**right**) of intraperitoneal insulin tolerance test (ITT). **D** (**top**), Heatmap of inflammatory cytokines abundance in gWAT measured by electrochemiluminescence-based assay (red=high, white=low). **Bottom**, Levels are shown as fold change (FC) compared with healthy control mice. Concentrations of IL-1β (interleukin-1β), IL-6 (interleukin-6), and TNF-α (tumor necrosis factor-α). **E** (**from left to right**), Schematic representation of HFpEF induction in C57BL/6JRj mice fed the HFpEF diet ad libitum for 24 weeks followed by weekly subcutaneous injections (s.c.) of anti-IL-1β or IgG control (0.5 mg/kg body weight; open and closed circles, respectively) for 5 weeks. Mice were then randomly divided into weight-matched groups (as left **B**). Body weight gain, gWAT weight/TL, LVEF, E/e’, and HW/TL are shown. **F**, Graphical abstract summarizing the hypothesis and main findings of the study. Unlike CR, which lowers body weight and adiposity through activating lipolysis across organs, ATGLi exerts its beneficial effects by inhibiting lipolysis primarily in adipose tissue. Data are presented as mean±SEM, except for body weight panels (mean±SD), with subject-level data superimposed. Statistical analysis was performed using GraphPad Prism (version 10.4.1). *P* values were calculated by factorial ANOVA (**A**, **B** [**left**], and **E**) with the following pairwise comparisons reported. In the case of body weight gain, the Greenhouse-Geisser–corrected repeated-measures mixed model was used. One-way ANOVA, Welch test, or Kruskal-Wallis were applied, as appropriate (in **B**, **C**, and **D**). Data residual distribution was examined by the Shapiro-Wilk test, whereas homogeneity of variance was verified by the Brown-Forsythe test. Indicated sample sizes adjacent to the *x*-axis in every panel or plot legend refer to biological replicates. Schemes and panels were created with BioRender.com licensed to the Medical University of Graz.

To enhance the translational potential of these observations, we examined the therapeutic efficacy of selective pharmacological inhibition of ATGL in AT—while sparing the heart to circumvent cardiac detrimental effects—using atglistatin (ATGLi).^5^ For this, male 7-week-old C57BL/6JRj mice were given a high-fat diet+L-NAME for 15 weeks before ATGLi treatment (Figure [B]). Because HFpEF+ATGLi mice ate on average 31% fewer calories than mice fed HFpEF diet ad libitum, we performed pair-feeding experiments to determine whether the cardiac effects of ATGLi stem solely from its impact on reduced food intake. In addition to these HFpEF+CR mice, a subset of HFpEF mice continued receiving high-fat diet+L-NAME ad libitum, serving as HFpEF controls. As compared with control HFpEF mice that continued to gain body weight, both HFpEF+ATGLi and HFpEF+CR mice displayed comparable body weight loss (Figure [B]). However, gWAT weight/tibia length was significantly lower in the HFpEF+ATGLi group than in the HFpEF+CR group, indicating that CR alone does not fully recapitulate the metabolic benefits of ATGLi on visceral adiposity (Figure [B]). Ejection fraction was preserved in all groups (Figure [B]). However, diastolic dysfunction and cardiac hypertrophy were improved in HFpEF+ATGLi and HFpEF+CR mice (Figure [B]), despite similar systolic blood pressure (not shown). Notably, ATGLi more effectively improved diastolic dysfunction than CR alone with a comparable reduction of left ventricular hypertrophy (Figure [B]). Glucose and insulin tolerance tests also revealed comparable improvements in glucose sensitivity and insulin resistance upon ATGLi treatment and CR (Figure [C]).

Next, we evaluated cytokine levels in gWAT to determine the role of AT-derived inflammation in mediating the anti-HFpEF effects of ATGLi (Figure [D]). We detected a robust decline in IL-6 (interleukin-6), IL-1β (interleukin-1β), and TNF-α (tumor necrosis factor-α) levels in both HFpEF+ATGLi and HFpEF+CR mice as compared with HFpEF controls. Interestingly, the decline in IL-1β levels was more pronounced with ATGLi, reflecting its superior adiposity-lowering and cardioprotective impact. Therefore, we subcutaneously injected mice once weekly with IL-1β neutralizing antibodies or IgG isotype for 5 weeks in the absence or presence of ATGLi to study the role of IL-1β inhibition in mediating the cardioprotective effects of ATGLi in HFpEF (Figure [E]). While chronic inhibition of IL-1β had no effects on body weight gain, gWAT mass, ejection fraction (EF), and left ventricular remodeling (Figure [E]), it significantly improved diastolic dysfunction in HFpEF mice. By contrast, IL-1β neutralization failed to further improve diastolic dysfunction in ATGLi- and CR-treated mice (Figure [E]). Collectively, these findings demonstrate that IL-1β is implicated in maladaptive AT‐heart communication in cardiometabolic HFpEF.

This study has limitations. The disproportionate reduction in gWAT weight between ATGLi and CR suggests that ATGLi may be superior in improving diastolic dysfunction due to its stronger anti-obesity effects. Although ANCOVA analysis does not support this interpretation, this cannot be ruled out in the absence of equivalent reductions in gWAT mass and body weight by ATGLi and CR alone. Achieving such equivalence would require more severe CR, which may produce adverse effects and limit translational relevance. Thus, it cannot be definitely excluded that body weight reduction may account for the therapeutic benefits of ATGL inhibition in HFpEF. Regardless, while reductions in adiposity and inflammation significantly contributed to the anti-HFpEF effects of ATGLi, additional mechanisms might be involved. Indeed, although anti–IL-1β antibodies failed to further enhance the cardioprotective effects of ATGLi, this does not prove that IL-1β inhibition alone is responsible for the observed benefits of ATGLi. Therefore, further investigation is warranted to establish causality between IL-1β and HFpEF. Future studies should also compare the anti-HFpEF efficacy of ATGLi with that of established treatments, such as SGLT2 inhibitors and GLP-1 receptor agonists. Moreover, the potential benefits of ATGLi in nonobese HFpEF phenotypes associated with aging and hypertension remain to be explored.

Notwithstanding these limitations, our study identifies AT lipolysis as a novel therapeutic target for obesity-related HFpEF, paving the way for future clinical trials evaluating the safety and efficacy of emerging AT-specific human ATGL inhibitors, such as NG-497, both as adjunctive treatments and potentially as standalone therapies for patients, who are intolerant or at higher risk of adverse effects from currently approved HFpEF pharmacotherapies.

## Data Availability

Data and materials supporting the results or analyses presented in this publication are available on reasonable request to the corresponding authors.

## ARTICLE INFORMATION

### Acknowledgments

The authors are grateful to Viktoria Trummer-Herbst and Tobias Huberts for excellent technical support, and to the staff members of the animal facilities at the Medical University of Graz and University of Graz. A. Stockner, D. Verma, S.T. Schmid, A. Fuerlinger, and A. Varghese are currently trained as PhD candidates in the PhD Program Molecular Medicine at the Medical University of Graz.

### Sources of Funding

This work was supported by the Program Molecular Medicine at the Medical University of Graz (to S. Sedej and A. Stockner, 2022–2025). S. Sedej, R. Schreiber, and M. Abdellatif are grateful to the Austrian Science Fund for the Cluster of Excellence MetAGE (10.55776/COE14). A. Stockner received an European Molecular Biology Organisation (EMBO) Scientific Exchange Grant and a Marietta-Blau Fellowship from the Austria’s Agency for Education and Internationalisation (Österreichs Agentur für Bildung und Internationalisierung [OEAD]).

### Disclosures

R. Breinbauer has filed a patent for human adipose triglyceride lipase inhibitors. The other authors report no conflicts.
